# Molecular biology of serotonergic systems in avian brains

**DOI:** 10.3389/fnmol.2023.1226645

**Published:** 2023-07-19

**Authors:** Toshiyuki Fujita, Naoya Aoki, Chihiro Mori, Koichi J. Homma, Shinji Yamaguchi

**Affiliations:** ^1^Department of Biological Sciences, Faculty of Pharmaceutical Sciences, Teikyo University, Tokyo, Japan; ^2^Department of Molecular Biology, Faculty of Pharmaceutical Sciences, Teikyo University, Tokyo, Japan

**Keywords:** serotonin, serotonergic system, serotonergic receptor, avian brain, homology

## Abstract

Serotonin (5-hydroxytryptamine, 5-HT) is a phylogenetically conserved neurotransmitter and modulator. Neurons utilizing serotonin have been identified in the central nervous systems of all vertebrates. In the central serotonergic system of vertebrate species examined so far, serotonergic neurons have been confirmed to exist in clusters in the brainstem. Although many serotonin-regulated cognitive, behavioral, and emotional functions have been elucidated in mammals, equivalents remain poorly understood in non-mammalian vertebrates. The purpose of this review is to summarize current knowledge of the anatomical organization and molecular features of the avian central serotonergic system. In addition, selected key functions of serotonin are briefly reviewed. Gene association studies between serotonergic system related genes and behaviors in birds have elucidated that the serotonergic system is involved in the regulation of behavior in birds similar to that observed in mammals. The widespread distribution of serotonergic modulation in the central nervous system and the evolutionary conservation of the serotonergic system provide a strong foundation for understanding and comparing the evolutionary continuity of neural circuits controlling corresponding brain functions within vertebrates. The main focus of this review is the chicken brain, with this type of poultry used as a model bird. The chicken is widely used not only as a model for answering questions in developmental biology and as a model for agriculturally useful breeding, but also in research relating to cognitive, behavioral, and emotional processes. In addition to a wealth of prior research on the projection relationships of avian brain regions, detailed subdivision similarities between avian and mammalian brains have recently been identified. Therefore, identifying the neural circuits modulated by the serotonergic system in avian brains may provide an interesting opportunity for detailed comparative studies of the function of serotonergic systems in mammals.

## 1. Introduction

Serotonin (5-hydroxytryptamine, 5-HT) is an ancient signaling molecule (Hay-Schmidt, 2000). Serotonin utilization is not only evolutionarily conserved in vertebrates and invertebrates of the animal kingdom; it has also been identified in plants (for further information, see the review by Erland et al., 2016). In the animal kingdom, numerous studies have suggested that the neuromodulatory functions of serotonin are implicated in a wide range of processes, including cognition, behavior, and emotion, and that these associations are also evolutionarily conserved (Jacobs and Azmitia, 1992; Lucki, 1998; Kandel et al., 2000; Bacque-Cazenave et al., 2020). In invertebrates, one of the most notable examples of the effects of serotonergic function on behavior is its effect on rapidly inducing notorious swarming and gregarious behavior in desert locusts (*Schistocerca gregaria*) (Anstey et al., 2009; Rogers and Ott, 2015). In vertebrates and especially humans, the serotonergic system has received much attention as a pharmacological target because it has been implicated in many brain functions associated with psychiatric disorders (Belmaker and Agam, 2008; Ravindran and Stein, 2010). Researchers have sought to understand, at the neural circuit level, the brain regions involved in mammalian cognitive, behavioral, and emotional processing, as well as the specific cells modulated by serotonin within these regions (Olivier, 2015; Strac et al., 2016; Yagishita, 2020). For example, the serotonergic system is implicated in the regulation of fear-related behaviors in mammals by modulating the neural microcircuits of the amygdala via several serotonergic receptors (LeDoux, 2000; Deakin and Graeff, 2013; Duvarci and Pare, 2014; Janak and Tye, 2015; Tovote et al., 2015; Bocchio et al., 2016; Lawther et al., 2020). However, it remains to be elucidated whether the neural circuits involved in cognitive, behavioral, and emotional processing, as well as their relationships with serotonergic modulation, are evolutionary conserved across vertebrates. To address this issue, it is necessary to clarify such neural circuits in a range of vertebrate animals other than mammals.

At the conceptual level, serotonergic systems display remarkable conservation across vertebrate species (Parent, 1981), suggesting evolutionary conserved roles in brain functions. However, the macro structure of vertebrate brains is diverse and lineage dependent, limiting the availability of information about which neural circuits are involved in cognition, behavior, and emotion (Pessoa et al., 2019). Birds represent unique model animals for elucidating the evolutionary continuity of the neural basis of cognition, behavior, and emotion because of the availability of information on several neural circuits associated with behaviors (Jarvis et al., 2005; Rosa Salva et al., 2015; Martinez-Garcia and Lanuza, 2018; Papini et al., 2019). As a result of the efforts of many scientific investigators over the years, the avian research community currently has access to a wealth of resources including behavior-related brain lesion data (Benowitz, 1980; Matsushima et al., 2003), comprehensive brain region projection relationships (Axer et al., 2016; Herold et al., 2019; Stacho et al., 2020), a revised consensus on brain nomenclature (Reiner et al., 2004), and several excellent brain atlases (e.g., for chicken, Kuenzel and Masson, 1988; Puelles et al., 2018).

In this review, we summarize the currently available information on avian brain serotonergic systems. This review consists of four sections. First, we briefly introduce well-studied serotonergic systems in the mammalian brain for comparison with those of birds. Second, we discuss the extent of homology between avian and mammalian brains. There is some controversy about the correspondence between bird and mammal brains, but here we focus on homologies. Third, we describe serotonergic systems in chicken brains, mainly based on our recent molecular dissection work in chicks (Fujita et al., 2019, 2020, 2022a,b,c). Fourth, we briefly summarize the functions of serotonergic systems in bird brains, focused at the level of gene function.

## 2. A brief introduction to serotonergic systems in mammals

There are two independent serotonergic systems in mammals. On the one hand, the central serotonergic system functions as a central neurotransmitter, whereas on the other the peripheral serotonergic system works as a peripheral signaling molecule. The proportion of serotonin contained in the central nervous system is small; most serotonin is located in the gut, where it contributes to important physiological functions such as motility, secretion, and vasodilation (Erspamer, 1937; Erspamer and Asero, 1952; Spohn and Mawe, 2017). Importantly, the central and peripheral serotonergic systems are completely independent, since serotonin molecules cannot cross the blood–brain barrier (Xu et al., 2014; Mosienko et al., 2020).

**Figure 1:**
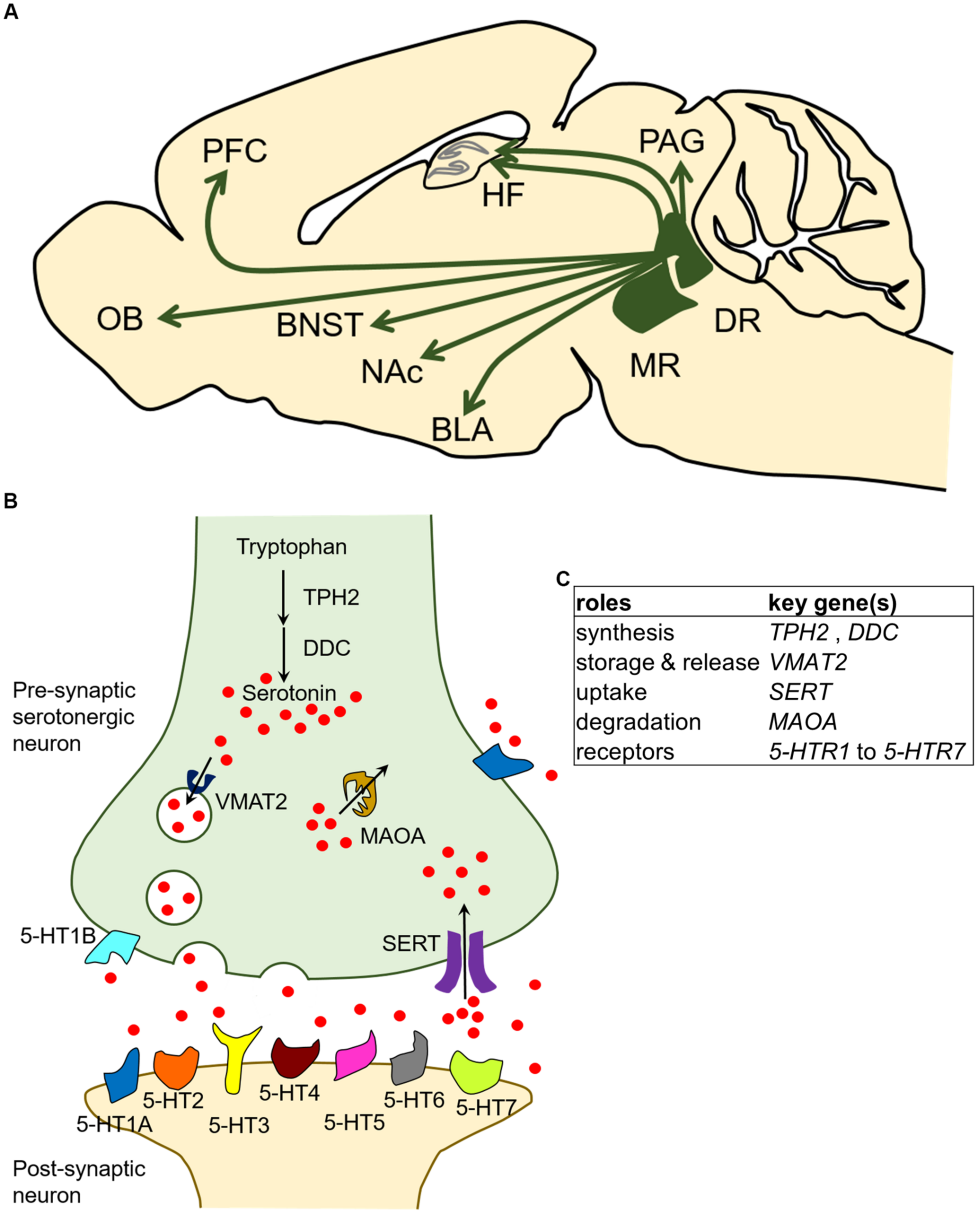
Localization and broad brain projections of the dorsal raphe and median raphe nuclei in the mammalian central serotonergic system and the molecular characteristics of serotonergic neurons. **(A)** Serotonergic neurons of the central serotonergic system are localized to the raphe nuclei of the brainstem, and the DR and MR send extensive projections throughout the forebrain and midbrain. The model diagram represents a sagittal section of a rodent, and it should be noted that the projected brain regions may not all be observable on the same sagittal plane. Arrows indicate the presence of projections. BLA, basolateral amygdala; BNST, bed nucleus of the stria terminalis; DR, dorsal raphe nucleus; HF, hippocampal formation; MR, median raphe nucleus; NAc, nucleus accumbens; OB, olfactory bulb; PAG, periaqueductal gray; PFC, prefrontal cortex. **(B)** In serotonergic neurons, serotonin is synthesized by the hydroxylation of tryptophan by tryptophan hydroxylase 2 (TPH2) followed by decarboxylation by DOPA decarboxylase (DDC). Serotonin is packaged into storage vesicles by vesicular monoamine transporter 2 (VMAT2) and released into the synaptic cleft. The released serotonin is mediated by serotonin receptors (5-HTR1 to 5-HTR7) present on the membranes of presynaptic and postsynaptic neurons, and signals are transmitted to cells. Serotonin in the synaptic cleft is taken up by serotonin transporter (SERT) and degraded by monoamine oxidase A (MAOA). **(C)** Summary table of characteristic genetic influences of serotonergic neurons schematized in panel **(B)**.

In the central nervous system, serotonergic neurons are present in cell groups in the raphe nuclei of the brainstem. Cell groups containing serotonergic neurons have historically been called groups B1–B9 (Dahlstroem and Fuxe, 1964; Soiza-Reilly and Gaspar, 2020). Among these serotonergic cell groups, the dorsal raphe (DR: B6 and B7) and median raphe (MR: B5 and B8) provide ascending innervation to the forebrain and midbrain (Figure 1A; Olivier, 2015; Cohen and Grossman, 2020; Soiza-Reilly and Gaspar, 2020). Given that the neural projections from the DR and MR cover almost all areas of the forebrain, including the amygdala, nucleus accumbens (NAc), bed nucleus of the stria terminalis (BNST), olfactory bulb (OB), prefrontal cortex (PFC), hippocampal formation (HF), and periaqueductal gray (PAG) (Figure 1A), and are involved in the regulation of many cognitive, behavioral, and emotional states and processes such as learning, reward, aggression, impulsivity, anxiety, and mood, the DR and MR serotonergic systems have been the subjects of intensive research (Olivier, 2015; Lawther et al., 2020; Liu et al., 2020; O'leary et al., 2020; Yagishita, 2020; Awasthi et al., 2021; Beyeler et al., 2021).

In the serotonergic neuron, serotonin is synthetized in a two-step process: conversion of the amino acid tryptophan (Trp) to 5-hydroxytryptophan (5-HTP), and subsequent decarboxylation of 5-HTP to 5-HT (serotonin). The first, rate-limiting step is mediated by tryptophan hydroxylase 2 (Tph2) (Walther et al., 2003; Walther and Bader, 2003). The second step is mediated by the aromatic amino acid decarboxylase (Aadc), also called DOPA decarboxylase (Ddc) because it is involved in dopamine biosynthesis. The synthesized serotonin is then stored in secretory vesicles by vesicular monoamine transporter 2 (Vmat2) and the vesicles later transport serotonin to the presynaptic terminal for release (Schuldiner et al., 1995). The serotonin released in the synaptic cleft is taken up by the presynaptic neuron through the serotonin transporter (Sert), which is encoded by the *solute carrier family 6 member 4* (*Slc6a4*) gene (Hoffman et al., 1991; Lesch et al., 1993). After the reuptake of serotonin into the serotonergic neurons, monoamine oxidase A (Maoa) catalyzes the oxidative deamination of 5-HT (Youdim et al., 2006). Among the proteins involved in serotonin synthesis, storage, release, uptake, and degradation, Ddc, Vmat2, and Maoa are involved in non-serotonin monoaminergic neuron processes and are thus expressed in both serotonergic and non-serotonergic neurons (Lein et al., 2007; Ng et al., 2009, 2010). In contrast, Tph2 and Sert are useful as molecular markers of serotonergic neurons because they function exclusively in the serotonergic neurons at least in the central nervous system of adult mammals. It should be noted that *Sert* is transiently expressed in non-serotonergic neurons in several brain regions during development (Lebrand et al., 1996; Narboux-Nême et al., 2008). In addition, the released serotonin acts on its downstream targets through multiple receptors called 5-HT receptors (5-HTRs) which exist on the cell membrane. To date, 14 distinct receptors have been identified and classified into seven groups, namely 5-HTR1 to 5-HTR7, based on their functional, structural, and biochemical characteristics. All 5-HTRs are G protein-coupled receptors (GPCRs) except for 5-HTR3 family members, which are ligand-gated ion channels (Hannon and Hoyer, 2008; Marin et al., 2020). An overview of the characteristic molecular mechanisms of serotonergic neurons described above is presented in Figures 1B,C. In terms of molecular components, the monoaminergic (including serotonergic) systems are thought to be largely conserved in vertebrates (Yamamoto and Vernier, 2011).

## 3. The avian brain

**Figure 2:**
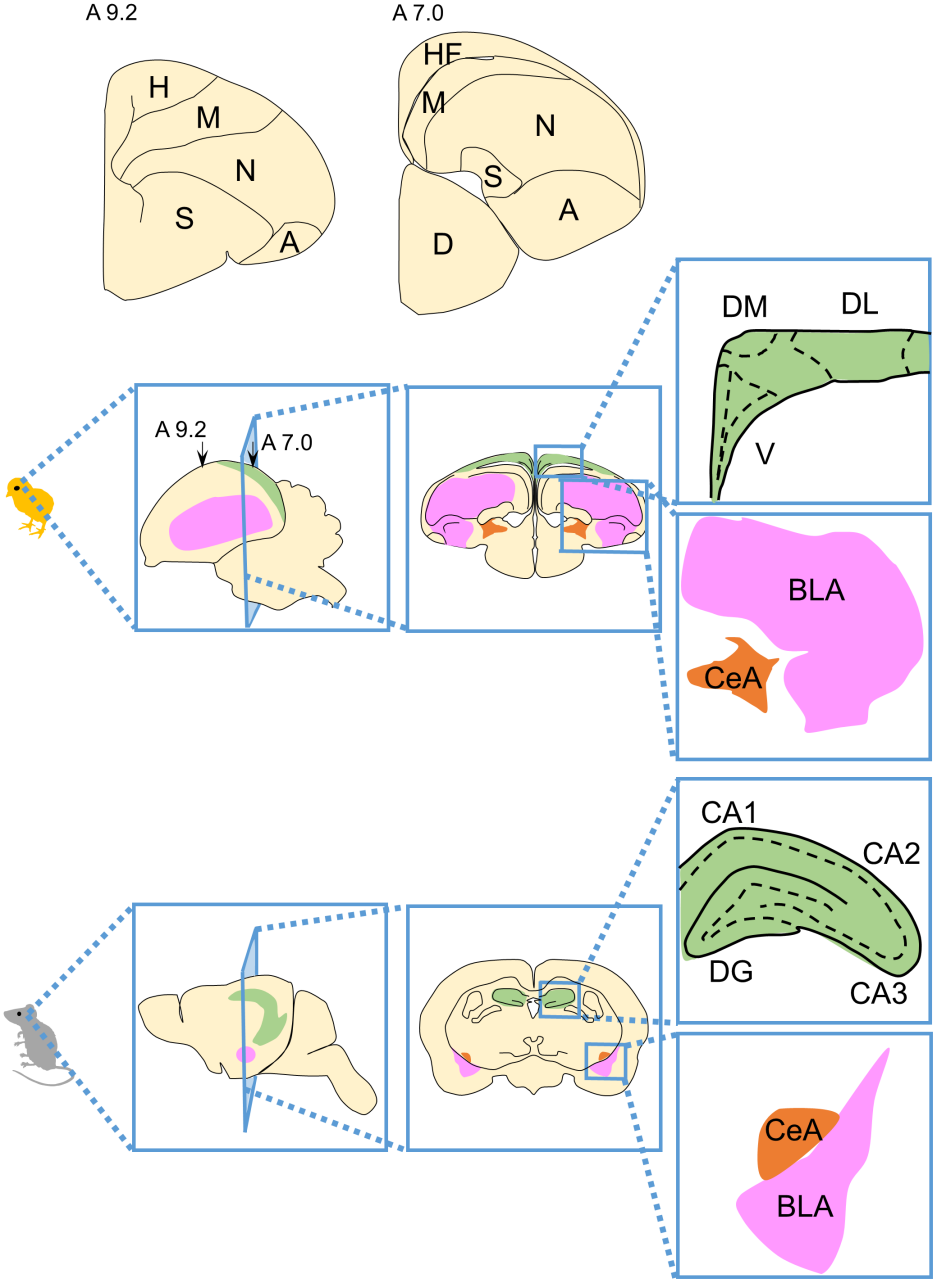
Schematic drawings of the structure of the avian telencephalon and its correspondence with the mammalian telencephalon. The avian telencephalon contains the pallium and subpallium. The largest portion of the pallium is the dorsal ventricular ridge (DVR) consisting of the mesopallium (M), nidopallium (N), and arcopallium (A). Schematic representations of hemisphere sections are shown above, with section levels according to the atlas of Kuenzel and Masson (1988). A schematic diagram of the brain structures of a chicken (as an example of an avian brain) and mouse (as an example of a mammalian brain) is shown. Portions considered to be homologous regions are indicated by the same color code. Green indicates the HF, magenta indicates the BLA, and orange indicates the CeA in the brains of both birds and mammals. A, arcopallium; BLA, basolateral amygdala; CA1, cornu ammonis field 1, CA2, cornu ammonis field2; CA3, cornu ammonis field 3; CeA, central nucleus of the amygdala; D, diencephalon; DG, dentate gyrus; DL, dorsal lateral region; DM, dorsal medial region; H, hyperpallium; HF, hippocampal formation; M, mesopallium; N, nidopallium; S, striatum; V, V-shaped complex.

The field of comparative neuroscience has revealed that the basic organization of the brain is shared across vertebrates. The vertebrate brain consists of the forebrain, midbrain, and hindbrain. Although the midbrain and hindbrain tend to be relatively well conserved, the forebrain has undergone major evolutionary changes. The telencephalon, which occupies most of the forebrain, can be divided into the pallium and subpallium (Striedter, 2005). In the avian pallium, the dorsal ventricular ridge (DVR), which consists of the mesopallium, nidopallium, and arcopallium, is a large proportion of the entire brain (Figure 2; Aboitiz et al., 2003; Molnár, 2011). The macro structure of the adult avian pallium looks quite different from that of mammals, making it difficult to understand its correspondence with the adult mammalian pallium, a historically controversial subject (Karten, 1997; Puelles, 2001; Jarvis et al., 2005). Regarding the macro structural differences between the adult pallium of birds and mammals, for example, while most mammalian cortex exhibits a six-layered structure, most superficial areas of avian pallium appear to lack an obvious layered structure and are instead organized into clusters of neurons (nuclei) (Karten, 2015). In order to investigate whether the neural circuits processing specific cognitive, behavioral, emotional processes are evolutionarily conserved or not, it is necessary to understand the homology of brain regions between birds and mammals.

One of the most commonly used methods to study homology is to compare expression regions of genes that are highly conserved in broad animal phyla during early development. Such research has led to the creation of a field referred to as evolutionary developmental biology (Evo-Devo), and one of the best-known discoveries in this field is that many of the key genes involved in developmental patterning, called ‘toolkit genes,’ are conserved across all bilaterally symmetric animal phyla. These toolkit genes consist of a small set of master regulatory genes, including transcription factors and signal transduction molecules (Carroll, 2008; Carroll et al., 2013). Studies have been performed to elucidate homologous regions of vertebrate pallium by combining expression regions of regulatory genes conserved in vertebrates in morphogenesis at early embryonic stages and morphological landmarks (Fernandez et al., 1998; Puelles et al., 2000). Currently, the embryonic pallium has been divided into four (Puelles et al., 2017) or six (Desfilis et al., 2018) components, it has been clarified that pallium derivatives correspond to adult brain regions. Here, we refer to the proposed pallium divisions as the medial, dorsal, dorsolateral/lateral, and ventral/ventrocaudal pallial divisions (Pessoa et al., 2019). The brain structures derived from each component of the pallium in birds and mammals have been proposed as follows: the medial pallium gives rise to the hippocampal formation in birds and mammals; the dorsal pallium gives rise to the hyperpallium in birds and the neocortex in mammals; the dorsolateral/lateral pallium give rise to the mesopallium in birds and the claustro-insular region, orbitofrontal cortex rostrally, and perirhinal/lateral entorhinal cortex caudally in mammals; and the ventral/ventrocaudal pallium give rise to the arcopallium and nidopallium in birds and the olfactory cortex and pallial amygdala, which is part of amygdala, in mammals (Puelles, 2001; Moreno and González, 2006; Medina and Abellán, 2009; Puelles et al., 2017; Medina et al., 2017a; Desfilis et al., 2018). Depending on the species, pallium-derived brain structures may undergo different developmental trajectories after late development and may acquire a different cytoarchitecture, neurochemical features, and connectivity (Puelles and Medina, 2002; Striedter, 2005; Medina et al., 2017a). Therefore, it is difficult to understand the homology of brain regions between different species by comparing the properties of the adult traits and terminal functional molecules alone. The results of recent single-cell RNA sequencing analyses strongly support the homologous relationship between the avian and mammalian brain regions described above, based on similarities such as the combinatorial profiles of transcription factors that determine cell properties (Tosches et al., 2018; Colquitt et al., 2021).

In addition, homologies are beginning to reveal correspondences between subdivisions of avian and mammalian brain structures. Here, we briefly discuss the current state of two structures recognized for their important contributions to cognitive, behavioral, and emotional processing: the hippocampal formation (HF) and amygdala (Figure 2).

We first discuss HF. All mammalian HFs are well conserved for macroscopic histological features (Hevner, 2016). They are composed of similarly complex and intertwined three-layered subdivisions, known as the Ammon’s horns or Cornu ammonis (CA) fields 1 (CA1), CA2, CA3, dentate gyrus (DG), and the subiculum (Hevner, 2016; Medina et al., 2017a). As a connection relationship within such subdivisions of HF, a neural circuit called “trisynaptic circuit” is considered to be important. The trisynaptic ciruit involves subdivisions of the entorhinal cortex (EC), DG, CA3, and CA1, and consists of projections from EC to DG, DG to CA3, and CA3 to CA1(Sloviter and Lomo, 2012). Among these projections, the DG to CA3 projection is called the “mossy fiber” and is a well known anatomical landmark of the HF. On the other hand, avian HFs occupy a large area of the caudal surface of the pallium with a high density of neurons, with a layered structure that is not readily observable macro-anatomically and subdivision that is similarly not clearly observable (Atoji and Wild, 2006; Herold et al., 2015; Striedter, 2016). Furthermore, the mossy fiber-like structures seen in the mammalian DG have not been found in avian HF (Faber et al., 1989; Montagnese et al., 1993, 1996; Tombol et al., 2000; Herold et al., 2014). To date, several subdivisions have been proposed for avian HFs, using several methods and criteria, such as histology, immunohistochemistry, projection relationships, and developmental origin (Kuenzel and Masson, 1988; Atoji and Wild, 2004, 2006; Suarez et al., 2006; Gupta et al., 2012; Abellan et al., 2014; Herold et al., 2014; Atoji et al., 2016; Medina et al., 2017a; Puelles et al., 2018). However, there is controversy over homologous regions within avian HFs for mammalian DG and CA fields, and it is difficult to say that a one-to-one correspondence between avian HFs and mammalian HFs have been established for subdivision within HFs (Atoji and Wild, 2004, 2006; Kempermann, 2012; Abellan et al., 2014; Herold et al., 2014; Atoji et al., 2016; Hevner, 2016; Striedter, 2016; Medina et al., 2017a). Briefly, there are three possibilities for homology with subdivisions of mammalian HFs for the subdivision of avian HFs. A first possibility is that the V-shaped complex (V) in the avian HF corresponds to the mammalian DG (Atoji and Wild, 2004; Suarez et al., 2006; Gupta et al., 2012; Herold et al., 2014; Atoji et al., 2016; Puelles et al., 2018). A second possibility is that the dorsal medial region (DM) in the avian HF corresponds to the mammalian DG (Montagnese et al., 1996; Szekely, 1999). A third possibility is that the avian HF does not have a homolog of the mammalian DG. The underlying idea is that it has been so long since birds and mammals diverged that it is difficult to compare HF subdivisions between birds and mammals in the first place. In other words, the idea is that the DG is a uniquely acquired trait of mammalian HF (Hevner, 2016; Striedter, 2016). Recently, neurons with a function similar to “place cells” that selectively fire when passing through a specific place have been identified in the HF of birds (Payne et al., 2021). In mammals, place cells are recognized as pyramidal neurons in the CA1 and CA3 as well as granule cells in the DG of the trisynaptic circuit (O'Keefe and Dostrovsky, 1971; Muller et al., 1987; Jung and Mcnaughton, 1993; Wilson and Mcnaughton, 1993). This finding raises the possibility that a neural circuit corresponding to the trisynaptic circuit exists in the HF of birds. The current state of the avian HF, especially in chickens, was recently reviewed (see Morandi-Raikova and Mayer, 2022).

We second discuss the amygdala. The mammalian amygdala is a brain region composed of a heterogeneous assembly of nuclei derived from both the subpallial nuclei and pallial nuclei. The amygdala has been investigated as one of the regions responsible for processing emotions, including learned fear (LeDoux, 2000; Medina et al., 2017b; Martinez-Garcia and Lanuza, 2018). Under laboratory conditions, the critical neural microcircuits responsible for learned fear have been revealed using Pavlovian fear conditioning paradigms. Among the subdivisions of the amygdala, the lateral nucleus (LA), basal nucleus (BA), and basomedial (BM) nucleus together constitute such a neural circuit with the involvement of the basolateral complex of the amygdala (BLA). In this circuit, the LA receives sensory projections from outside of the amygdala and projects to the BA, which projects to the central nucleus of the amygdala (CeA) to provide output to the brainstem (LeDoux, 2000; Duvarci and Pare, 2014; Janak and Tye, 2015; Tovote et al., 2015).

On the other hand, it has been proposed that regions homologous to the avian pallial amygdala are the arcopallium and nidopallium, as described above, but there is no consensus among the avian research community.

Historically, the homologous region of the avian pallial amygdala has often been referred to as the arcopallium, and in recent years the avian amygdala tends to be treated as the arcopallium/amygdala complex, which combines the arcopallium, posterior pallial amygdala, nucleus taeniae of the amygdala (TnA), and subpallial amygdaloid area (Herold et al., 2018). Regarding the nidopallium, previous studies have functionally associated it with the mammalian PFC, so few attempts have been made to associate the nidopallium with the mammalian amygdala (Karten, 1997, 2013; Güntürkün, 2005; Butler et al., 2011; Dugas-Ford et al., 2012; Herold et al., 2018). Although no previous studies using the Pavlovian fear conditioning paradigm have been conducted to examine the functions involved in the arcopallium and nidopallium, several studies using the pigeon appetite conditioning paradigm have been conducted to implicate parts of the arcopallium and nidopallium in extinction learning (Lissek and Güntürkün, 2005; Lengersdorf et al., 2014, 2015; Starosta et al., 2017; Gao et al., 2018, 2019a,b; Güntürkün et al., 2020). In avian, the medial and central amygdaloid structures and output projections from amygdala have been unveiled (Abellán and Medina, 2009; Vicario et al., 2014; Hanics et al., 2017).

In summary, the crude homology between avian and mammalian pallium is grossly apparent. However, the correspondence between brain structural subregions remains unclear due to the divergence of adult brain morphology. In the future, the development of single-cell transcriptomics that traces the developmental processes of avian pallium will clarify the homology between the brain subregions of birds and mammals. In addition, a future task is to clarify the extent to which neural circuits between homologous regions of the brains of birds and mammals share common cognitive, behavioral, and emotional functions.

## 4. Molecular dissections of the serotonergic system in the chick brain

The central serotonergic system of vertebrates has a conserved form in which serotonergic neurons reside in the raphe nuclei of the brainstem, where the DR and MR send neuronal projections throughout the forebrain and midbrain (Parent, 1981). The distribution of serotonergic neurons in the central serotonergic system of vertebrates other than mammals has been identified to an extent. In fish, for example, the locations and projections of serotonergic neurons have been comprehensively described by generating a transgenic zebrafish line expressing GFP under control of the regulatory elements of *pet1*, referred to as the serotonergic neuron specific marker gene of the raphe (Lillesaar et al., 2009). In addition, the distribution and function of serotonergic neurons in fish have already been reviewed (Lillesaar, 2011). In amphibians, the organization of central serotonergic neurons has been described in several species, including salamander (Dubé and Parent, 1982; Clairambault et al., 1994; Dicke et al., 1997) and frog (van Mier et al., 1986; Bhat and Ganesh, 2023). In reptiles, the location of serotonergic neurons has been described in several species, such as turtle (Ueda et al., 1983), lizard (Smeets and Steinbusch, 1988), snake (Challet et al., 1991), and crocodile (Rodrigues et al., 2008). In birds, the distribution of serotonergic neurons has been described in multiple species, including chicken (Ikeda and Goto, 1971; Dube and Parent, 1981; Parent, 1981; Yamada, 1984), pigeon (Fuxe and Ljunggren, 1965; Challet et al., 1996; Meneghelli et al., 2009), and quail (Cozzi et al., 1991). In the vertebrates examined previously, the presence of serotonergic neurons as cell groups in the brainstem, specifically the DR and MR, has been confirmed. However, for many non-mammalian vertebrates, the anatomical information is not well organized, and hence the one-to-one correspondence with mammalian nuclei is not always obvious. In addition, to the best of our knowledge, detailed properties such as the molecular characteristics of the central serotonergic system neurons of non-mammalian vertebrate remain largely unknown. Furthermore, our understanding of the distribution of neurons that are modulated by serotonin (that is, those expressing *5-HTR*s) in non-mammalian vertebrae is limited. Therefore, using the chicken (*Gallus gallus domesticus*) as an avian model, which is easy to manage, we attempted to molecularly elucidate the central serotonergic system of the DR and MR. Below, we summarize the details of the distribution of serotonergic neurons in the DR and MR and the molecular heterogeneity of serotonergic neurons (Fujita et al., 2022b). We also summarize the expression regions of most *5-HTR* genes in the telencephalon (Fujita et al., 2020, 2022a), and the potential for serotonergic regulation of the dopaminergic system (Fujita et al., 2022c). All data presented below are based on results from post-hatched day 1 (P1) chick brain samples in controlled growing conditions (Yamaguchi et al., 2008a,b). The histological terminology used in these studies, and the histological atlas primarily used, is the terminology of the avian brain nomenclature consortium (Reiner et al., 2004), and the atlas of Kuenzel and Masson (1988), respectively. In addition, the atlas of Puelles et al. (2018) was also used for the histological position of several brain structures.

### 4.1. The distribution and heterogeneity of the serotonergic neurons in the chick DR and MR

Previous studies have described the anatomical position of the raphe nuclei (Kuenzel and Masson, 1988; Puelles et al., 2018), the distribution of monoamines based on fluorescence histochemistry (Ikeda and Goto, 1971; Dube and Parent, 1981; Parent, 1981), and the distribution of serotonergic neurons based on immunohistochemistry using an anti-serotonin antiserum (Yamada, 1984), as well as information about their developmental processes (Wallace, 1985; Sako et al., 1986; Okado et al., 1992; Huang X. et al., 2019) in chicken. Several studies have used chickens to describe the distribution of the brainstem cell groups containing cells with characteristics of serotonergic neurons. In addition, based on their anatomical position, possible correspondences between these chick cell groups and mammalian serotonergic cell groups have been proposed (Ikeda and Goto, 1971; Dube and Parent, 1981; Yamada, 1984). However, in the above-mentioned studies, the serotonergic cell groups were without consideration of the mammalian serotonergic cell groups, and hence, the group numbers do not necessarily match between mammals and birds. Therefore, we used the terms chicken DR and MR as follows: the DR for “B5” (Ikeda and Goto, 1971), “Group 5” (Yamada, 1984), and “DR nucleus” (Puelles et al., 2018); and the MR for “B6” and “B7” (Ikeda and Goto, 1971), “Group 6” and “Group 10” (Yamada, 1984), “rhombomere 1 median raphe nucleus (r1MnR),” “rhombomere 2 median raphe nucleus (r2MnR),” and “caudal linear nucleus of the raphe (CLi)” (Puelles et al., 2018).

First, the distribution of cell bodies of serotonergic neurons in the brainstem, including the chicken DR and MR, was analyzed using *in situ* hybridization (ISH) to determine the gene expression distribution of the chicken orthologues of the mammalian serotonergic neuron markers *TPH2* and *SERT* (Fujita et al., 2022b). *TPH2-* and *SERT*-expressing cells were found to be densely distributed in a lateral spread form from the mesencephalon to the medulla oblongata in the DR (equivalent to A2.0 to A1.0 in the Kuenzel and Masson Atlas). In the MR, they were found to be distributed slightly more towards the mesencephalon side than the DR (equivalent to A2.4 to A1.0 in the Kuenzel and Masson Atlas) from the mesencephalon to the medulla oblongata (Fujita et al., 2022b). A comparison of the expression patterns of these genes using serial sections of the brainstem showed a well-matched distribution (Fujita et al., 2022b), which corresponded well with the distribution of serotonergic neurons described in previous studies (Ikeda and Goto, 1971; Dube and Parent, 1981; Yamada, 1984), suggesting that these two genes are useful markers for specifically visualizing serotonergic neurons in chick brains. There is considerable heterogeneity among serotonergic neurons between and within the DR and MR in relation to multiple aspects, including their developmental origin, connectivity, electrophysiological properties, molecular organization, and behavioral functions in mammals (Calizo et al., 2011; Alonso et al., 2013; Okaty et al., 2019, 2020; Ren et al., 2019; Huang K. W. et al., 2019; Soiza-Reilly and Gaspar, 2020). In addition, developmental and projection target heterogeneity of serotonergic neurons also exist in fish (Gaspar and Lillesaar, 2012), suggesting that the existence of heterogeneity in serotonergic neurons is evolutionarily conserved in vertebrates. As an example of the molecular heterogeneity of serotonergic neurons, such neurons are known to express *5-HTR*s on their own and are subject to serotonergic modulation (Beyeler et al., 2021; De Deurwaerdere and Di Giovanni, 2021). There is heterogeneity between and within the DR and MR in which *5-HTR* is expressed in each serotonergic neuron (Okaty et al., 2019, 2020; Ren et al., 2019; Huang K. W. et al., 2019).

Moreover, the expression of most *5-HTR* orthologues was examined in the chick DR and MR. This revealed that *5-HTR1A, 5-HTR1B, 5-HTR1D, 5-HTR1E*, and *5-HTR5A* were differently expressed in chick DR and MR serotonergic neurons with partial overlap, indicating the heterogeneity in chick serotonergic neurons (Fujita et al., 2022b). Furthermore, the set of *5-HTR*s expressed in the serotonergic neurons of chicken DR and MR is similar to that of the mammalian DR and MR, suggesting that the molecular organization of serotonergic neurons is evolutionarily conserved. The exception is that the set of *5-HTR*s in the genome is not strictly identical from species to species, notable examples are the absence of orthologues in the genome: *5-HTR5B* is absent from the chicken genome (International Chicken Genome Sequencing Consortium, 2004) and *5-Htr1e* is absent from the mouse genome (Vilaró et al., 2020). Evolutionary conservation of the molecular organization of serotonergic neurons is also supported by studies showing similarities in gene expression during brainstem development in chickens and mice (Cambronero and Puelles, 2000; Alonso et al., 2013; Watson et al., 2019). In the future, further research is needed to clarify the heterogeneity, including projection destination preferences and their electrophysiological heterogeneity, in avian DR and MR serotonergic neurons in order to better understand the degree of conservation of the serotonergic system.

### 4.2. Expression of *5-HTR*s in the chick telencephalon

**Figure 3:**
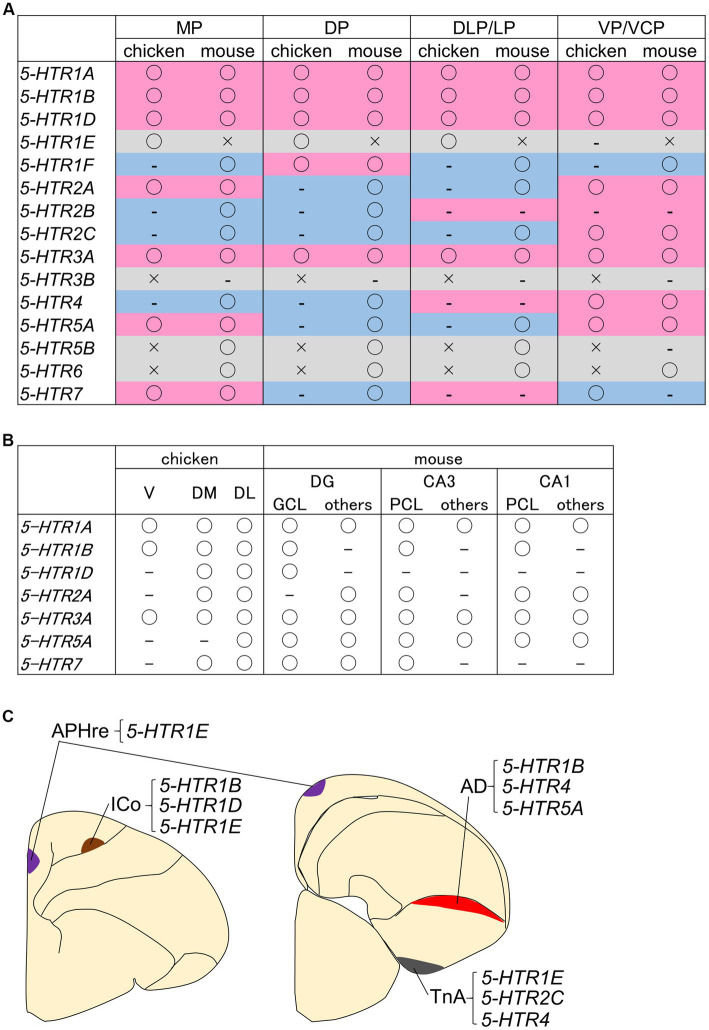
Summaries of *5-HTR*s expression information in the chicken pallium. Tables summarizing the pallium **(A)** and HF subdivisions **(B)** in context of whether the *5-HTR* family genes (and their orthologs) are present in chicken and mouse genomes. The brain structures derived from each pallium is as follows: the MP gives rise to the HF in birds and mammals; the DP gives rise to the hyperpallium in birds and the neocortex in mammals; the DLP/LP gives rise to the mesopallium in birds and the claustro-insular region, orbitofrontal cortex rostrally, and perirhinal/lateral entorhinal cortex caudally in mammals; and the VP/VCP gives rise to the arcopallium and nidopallium in birds and the olfactory cortex and pallial amygdala in mammals (Puelles, 2001; Moreno and González, 2006; Medina and Abellán, 2009; Puelles et al., 2017; Medina et al., 2017a; Desfilis et al., 2018). Please refer to the main text for more details on pallium homology. “○” indicates that it has been detected, “–” indicates that it has not been detected, and “×” indicates that it does not exist in the genome or has not been examined. These data are based on Fujita et al. (2020, 2022a) for chickens and on expression information from previous studies, including parts confirmed using the Allen brain atlas (Lein et al., 2007; Ng et al., 2009, 2010; Tanaka et al., 2012; Luo et al., 2019; Vilaró et al., 2020). The background colors in the table of panel **(A)** represent the following: magenta, common detection or non-detection in chicken and mouse; blue, divergent results between chicken and mouse; and light gray, comparison not possible because an “×” is included in one of the genomes. Note that the data for chickens were obtained by the chemical coloring *in situ* hybridization method, and the detection methods for mice are not necessarily the same. (**C**) Schematic representation of neuronal information with characteristic combinations of *5-HTR*s expression in the chicken telencephalon. Purple indicates APHre, brown indicates ICo, red indicates AD, and gray indicates TnA. AD, dorsal arcopallium; APHre, ectopic part of the rostral area parahippocampalis; CA1, cornu ammonis field 1, CA3, cornu ammonis field 3; DG, dentate gyrus; DL, dorsal lateral region; DLP/LP, dorsolateral pallium/lateral pallium; DM, dorsal medial region; DP, dorsal pallium; GCL, granule cell layer; ICo, intercalated core nucleus; MP, medial pallium; PCL, pyramidal cell layer; TnA, nucleus taeniae of the amygdala; V, V-shaped complex; VP/VCP, ventral pallium/ventrocaudal pallium.

Little is known about the distribution of neurons that are modulated by serotonin in the avian telencephalon. To date, for example, the site of action of 5-HTR1A in the pigeon brain has been studied using the selective radioligand [^3^H]-8-hydroxy-2-(di-n-propylamino)tetralin ([3H]-8-OH-DPAT) for 5-HTR1A (Dietl and Palacios, 1988; Waeber et al., 1989; Herold et al., 2012, 2014; dos Santos et al., 2015; Herold et al., 2018). We have previously comprehensively examined the expression regions of the above *5-HTR* orthologues in the chick whole telencephalon (Fujita et al., 2020, 2022a). Notably, the strongest and most widespread signals, including strong signals in the striatum, were obtained from *5-HTR1B*, while *5-HTR1A* and *5-HTR3A* had a sparse distribution of expressing cells in all brain regions examined. These expression patterns appear to be similar to those characteristic of *5-HTR1A*, *5-HTR1B*, and *5-HTR3A* expression patterns in the mouse brain (Lein et al., 2007; Ng et al., 2009, 2010). Therefore, in order to compare the gross characteristics of the brain regions where serotonin regulation occurs in birds and mammals, we summarized the *5-HTR* expression regions in the chick brain and existing information about *5-HTR* expression data in mice (Lein et al., 2007; Ng et al., 2009, 2010; Vilaró et al., 2020). A comparison of the information with a focus on homologous regions is shown in Figure 3A. From the ventral pallium/ventrocaudal pallium-derived brain structures, the arcopallium and nidopallium in chicks and the olfactory cortex and pallial amygdala in mice showed the most commonality in the presence or absence of the detection of *5-HTR* family genes. This fact is consistent with the widely accepted fact that the avian arcopallium is homologous and functionally conserved with part of the mammalian pallial amygdala (Medina et al., 2017b; Martinez-Garcia and Lanuza, 2018), and suggests that there are molecular commonalities in serotonergic regulation between birds and mammals. However, for DP-derived brain structures, the hyperpallium in chicks and neocortex in mice, opposite features were observed (Figure 3A). For example, the orthologues of *5-HTR2A* and *5-HTR2C* have characteristic expression patterns in the mammalian neocortex; however, no significant signal was detected in the chick hyperpallium (Fujita et al., 2020). Given that the neocortex is a highly specialized brain structure only in mammals, such a result does not deserve to be surprising. On the other hand, there is a consensus about the homology of the MP-derived HFs in both birds and mammals (Reiner et al., 2004; Herold et al., 2015; Striedter, 2016), and the ancient origin of HFs has been strongly corroborated by tissue dissected bulk transcriptome study (Belgard et al., 2013) and recent single-cell transcriptome study (Tosches et al., 2018). We compared the functions of each subdivision between avian and mammalian HFs from the viewpoint of the molecular basis of serotonin regulation, but there was no clear correspondence (Figure 3B).

In the mammalian HF, a large number of glutamatergic excitatory neurons are distributed in the DG granule cell layer, CA3 pyramidal cell layer, and CA1 pyramidal cell layer, which have high cell densities, and it is widely recognized that neuron populations in these layers constitute a trisynaptic circuit (Sloviter and Lomo, 2012; Hevner, 2016; Medina et al., 2017a). On the other hand, in general and except for developmental processes, cell types in avian HFs are not well understood at the molecular level, making it difficult to compare cell types with mammalian cell types. Some neuronal markers, such as calbindin, CaMKII, and DCX, which are used from a neurochemical point of view and in immunohistochemistry, are also used in the avian HF (Suarez et al., 2006; Melleu et al., 2013; Herold et al., 2019; Rook et al., 2023). A particularly interesting study using HF cell type markers by Atoji et al. (2016) showed that the orthologue of the DG granule cell population marker, *prox1*, was not only available as a developmental chicken DG region marker (Gupta et al., 2012; Abellan et al., 2014), but was also expressed in adult pigeon V. Since the avian V has been proposed to be equivalent to the mammalian DG (Atoji and Wild, 2004; Suarez et al., 2006; Gupta et al., 2012; Herold et al., 2014; Atoji et al., 2016; Puelles et al., 2018), this raised the possibility that *PROX1* could also be used as a marker for corresponding cells in the DG granule cell population marker in adult birds. To better understand the characteristics of serotonin-regulated neural circuits in the avian HF, it is necessary to increase the available markers for cell types within the avian HF. In the mouse HF, the *5-HTR1B* gene is selectively and strongly expressed in the CA1 pyramidal layer (Lein et al., 2007; Ng et al., 2009, 2010), whereas in the chick HF, *5-HTR1B*-expressing cells are sparsely distributed in the V and DM, and abundantly distributed throughout the DL (Fujita et al., 2020, 2022a). For example, if avian HF *5-HTR1B*-expressing cells can be cell typed, it may be possible to identify the distribution of neurons in the avian HF that share similarities in serotonergic control with mammalian CA1 pyramidal neurons.

In addition, several brain regions (nuclei) that exhibit characteristic *5-HTR*s expression patterns in the chick have been identified. First, the intercalated core nucleus (ICo) of the hyperpallium strongly expresses *5-HTR1B*, *5-HTR1D*, and *5-HTR1E* (Fujita et al., 2020, 2022a; Figure 3C). The ICo has been recently named by Puelles et al., 2018 to describe the nucleus, first noted for its zinc-rich property (Faber et al., 1989) and subsequently noted to express of *ENC1* (García-Calero and Puelles, 2009), *ZENK* (Zapka et al., 2010), and *NR4A2* genes (Puelles et al., 2016; Fujita et al., 2019). Since there is little information on how the ICo is in involved in brain functions such as cognition, behavior, and emotion, it is expected that attention will focus on serotonergic regulation prior to advances in our understanding. Second, *5-HTR1E* was found to be highly selectively expressed in a part of the DM subdivision of the HF, an ectopic part of the rostral area parahippocampalis (APHre) characterized by *LEF1* expression (Abellan et al., 2014; Figure 3C). LEF1 is a transcription factor required for the generation of granule cells in the mammalian DG (Galceran et al., 2000). A significant *LEF1*-expressing population during the development of HFs is commonly observed in both chickens and mice (Gupta et al., 2012; Abellan et al., 2014). However, in the adult HF, there is a significant *LEF1*-expressing population in the APHre in post-hatched chicken, whereas *LEF1* expression is not significant in the adult HF of mice (Lein et al., 2007; Ng et al., 2009, 2010). As such, the APHre may represent a unique cellular population within birds and be a region key to understanding the functional correspondence between avian and mammalian HFs. In chickens, the projection relationship of the APHre and related functions are completely unknown, but in the HF of pigeons, several excellent descriptions of the intra-HF projection relationships have been published (Atoji and Wild, 2004, 2006; Atoji et al., 2016; Herold et al., 2019; Rook et al., 2023). The subdivision chicken APHre (Abellan et al., 2014) appears to correspond to the parvocellular hippocampal area (Pa) (Atoji and Wild, 2004) or the dorsal dorsomedial (DMd) region (Herold et al., 2014) in pigeons, but detailed correspondence information remains to be elucidated. Confirming whether the Pa or DMd in pigeons and the APHre in chickens can be regarded as homologs and unifying terminology will be the first step toward understanding the functions of the APHre in birds. Third, *5-HTR1B*, *5-HTR4*, and *5-HTR5A* are expressed in the dorsal arcopallium (AD), while *5-HTR2C*, *5-HTR4*, and *5-HTR1E* are enriched in the TnA (Figure 3C). Previous studies on the distribution of serotonergic projections and terminals using anti-serotonin antiserum or antibody have shown that the AD and TnA of avian brains, including chickens (Yamada and Sano, 1985; Metzger et al., 2002) and pigeons (Challet et al., 1996), were commonly enriched with serotonergic nerve terminals. However, it was revealed that the AD and TnA express different *5-HTR* family genes, and focusing on the differences in their molecular compositions will help clarify the mode of serotonergic regulation that the AD and TnA receive.

Taken together, our previous work provided a comprehensive overview of *5-HTR* gene expression patterns in the chick telencephalon (Fujita et al., 2020, 2022a). For example, mammalian *5-Htr4* is known to have multiple splice variants (Bockaert et al., 2006; Marin et al., 2020), and it has been clarified that the expression site differs depending on the variant (Vilaró et al., 2005). As multiple isoforms of chicken *5-HTR4* are registered in the database, it is possible that there are differences in the expression site for each of these isoforms. To make a more detailed molecular comparison of serotonergic regulation in mammalian and avian brains, it is important to increase the information on the expression distribution of avian receptors. However, *5-HTR*s for whom characteristic expression patterns have not been clarified may exhibit low levels of expression. Therefore, in order to elucidate the detailed distribution, it may be necessary to use highly sensitive methods, such as using radioisotopes. Few prior studies have demonstrated the existence of the neural circuits involved in cognitive, behavioral, and emotional functions in bird brain regions. Information on the expression population of *5-HTR* genes provides a useful molecular basis for studying the role of neural circuits in birds in the future.

### 4.3. Serotonergic modulation of the other modulatory system in chicks

In addition to the central serotonergic system, the dopaminergic system is another important modulatory system known to be involved in motivation-related behavior. In addition, the central serotonergic system and the dopaminergic system interact with each other in mammals (Boureau and Dayan, 2011; De Deurwaerdere and Di Giovanni, 2017, 2021; Yagishita, 2020; Beyeler et al., 2021; Peters et al., 2021). The midbrain dopaminergic nuclei receive the projections from the DR including serotonergic neurons (Ogawa et al., 2014; Beier et al., 2015; Ogawa and Watabe-Uchida, 2018). Furthermore, recently, it has been clarified how dopaminergic neurons undergo serotonergic regulation at the neural circuit level and which *5-HTR*s are involved in mice (Wang et al., 2019; Peters et al., 2021). Interaction between the serotonergic and dopaminergic systems appears to occur in birds (Matsunami et al., 2012), but the molecular basis of this interaction was previously unknown. Through comprehensive examination of the expression of the *5-HTR*s probe set described above, we revealed that *5-HTR1A* and *5-HTR1B* are expressed in chick dopaminergic nuclei (Fujita et al., 2022c). Therefore, it was suggested that the dopaminergic system is also regulated by serotonin through *5-HTR1A* and *5-HTR1B* in birds, supporting the importance of the interaction between the serotonergic and dopaminergic systems. Unlike in mammals, the *5-HTR1A*- and *5-HTR1B*-expressing cells and dopaminergic neuron marker expression did not overlap, and *5-HTR*s expressed directly in dopaminergic neurons could not be detected. It would be interesting to clarify serotonergic regulation of dopaminergic neurons in birds using methods with higher detection sensitivity and through pharmacological examination of dopaminergic neurons. Analysis of the molecular basis of interactions between modulatory systems in birds is nascent, and further analysis is required in the future.

## 5. Central serotonergic system functions in birds

The central serotonergic system in mammals has been demonstrated to be involved in a wide variety of cognitive, behavioral, and emotional processes, including mood, fear, anxiety, appetite, aggression, impulsivity, reward and learning (Jacobs and Azmitia, 1992; Lucki, 1998; Yagishita, 2020). These neuromodulatory functions of serotonin have also been investigated in birds. For example, behavioral-pharmacological studies have revealed the involvement of serotonergic regulation in aggression in pigeons (Fachinelli et al., 1989) and foraging behavior in chickens (Matsunami et al., 2012). In addition, associations with feather-pecking behavior, during which chickens on a poultry farm peck their feathers together sometimes resulting in cannibalism in extreme cases (Savory, 1995), have also been examined through combining analyses of behavior and brain serotonin levels (Kops et al., 2017). For an excellent review on the relationship between feather-pecking behavior and the serotonergic system the reader is referred to De Haas and Van Der Eijk (2018).

Although specific gene functions in the serotonergic system are not clearly linked to specific cognitive, behavioral, and emotional processes in birds, studies are beginning to focus on expression of the *5-HTR* genes. For example, a previous study linked high expression of *5-HTR2C* in the right cerebral hemisphere with susceptibility to being a victim of feather pecking (Yao et al., 2017), and another study correlated the expression levels of *5-HTR2A* and *5-HTR2B* in the caudal region of the left telencephalon with individual variations in cognition using reversal learning (Boddington et al., 2020). Association studies between *SERT* polymorphism and behaviors, of which there are a relatively large number, are now discussed.

In humans, the promoter region of the *SERT* gene has a repetitive sequence insertion/deletion (IN/DEL) polymorphism, known as the *SERT* polymorphic region (SERTPR, or *5-HT transporter* (*5-HTT*)-linked polymorphic region, 5-HTTLPR) that affects its expression level; it has been repeatedly indicated that this polymorphism is associated with many psychiatric states such as depression, anxiety, and suicidal behavior (Serretti et al., 2006; Canli and Lesch, 2007; Murphy et al., 2008). In chickens, a functional polymorphism considered analogous to human SERTPR has been discovered and shown to have associations to body weight gain and locomotion activity (Phi-Van et al., 2014). Subsequently, the same chicken SERTPR polymorphism was shown to be associated with feed intake (Kjaer and Phi-Van, 2016) and fear-related behaviors (Krause et al., 2017, 2019; Phi Van et al., 2018; Table 1). However, it should be noted that the behavioral assays used to measure fear-related behaviors in chickens have not been standardized and are not necessarily widely supported, unlike the common assay systems used in mammals for fear conditioning (LeDoux, 2000) and anxiety-related behaviors (reviewed in Allsop et al., 2014; O'leary et al., 2020). As a notable example, tonic immobility (TI), a paradigm often chosen in behavioral studies to observe indicators of fear in chickens, is thought to represent a form of reproducible hypnotic state (Gallup et al., 1971; Gallup, 1974). The bird TI has a long history of being used as an index of fear and, since it can be observed with good reproducibility, it is undoubtedly highly useful as an indicator of certain behaviors (Gallup, 1973; Fureix and Meagher, 2015). However, it is unclear whether the TI in birds should be compared to fear-related behavior in mammals. Elucidating the neural circuits that control TI in chickens will advance what kind of behavioral and emotional states TI represents, and thus what emotional states it should be compared to in mammals. In addition, there are also interesting examples of association studies combining the genotyping of *SERT* polymorphisms and ecological observations as well as behavioral tests in wild birds. Several *SERT* polymorphisms have been associated with behavioral traits, such as the performance of novel object tests and urban or rural habitat differences in great tits (*Parus major*) (Riyahi et al., 2015, 2022; Timm et al., 2018, 2019; Grunst et al., 2021; Thys et al., 2021), blackbirds (*Turdus erula*) (Müller et al., 2013), and dunnocks (*Prunella modularis*) (Holtmann et al., 2016), and have been linked to animal personalities such as boldness and aggression (Table 1). However, associations between *SERT* polymorphisms and behavioral traits have not always yielded consistent results across birds and populations. For example, no association was detected between *SERT* polymorphisms and behavioral tests using Seychelles warbler (*Acrocephalus sechellensis*) (Edwards et al., 2015), no SERT polymorphism was detected with black swans (*Cygnus atratus*) (Van Dongen et al., 2015), and when using great tits Timm et al. (2019) and Thys et al. (2021) had conflicting results regarding female aggression. The discrepancies in these results appear to be at least in part due to differences in species and populations, as well as the difficulty in collecting a sample size that enhances statistical power, due to the use of wild animals. More research in the field is required to facilitate a future meta-analysis that could elucidate the issue.

**Table 1:** Overview of association studies between *SERT* polymorphisms and physiology and behavior in birds.

Avian species	Types of polymorphism	Effect on physiology and/or behavior	References
Chicken	IN/DEL in promotor region	Gender-dependent body weight gain and locomotor activity	Phi-van et al. (2014)
*Gallus gallus*		Feed intake	Kjaer and Phi-van (2016)
		Open field test and light–dark test	Krause et al. (2017)
		Avoidance test in newly hatched chick and arousal test	Phi Van et al. (2018)
		Tonic immobility test	Krause et al. (2019)
	*SERT* paralog (*slc6a4b*) VNTR	Impulsive behavior	Abe et al. (2013)
Great tit	SNP in promotor region	Novel object test	Riyahi et al. (2015)
*Parus major*	SNPs in exon 3 and exon 8	Novel object test, breeding parameters	Timm et al. (2018)
	SNP in exon 1	Hissing behavior in females	Timm et al. (2019)
	SNPs in exon 12 and exon 13	Variation in hissing behavior in females	Thys et al. (2021)
	SNP in exon 6	Obstacle removal test in females	Grunst et al. (2021)
	SNP in promotor region	Novel object test, distress calling rate, and hissing behavior in females	Riyahi et al. (2022)
Blackbird			
*Turdus erula*	Microsatellite in exon 1	Urban or rural habitat type	Müller et al. (2013)
Dunnock	Microsatellite in exon 1	Flight-initiation distance in females	Holtmann et al. (2016)
*Prunella modularis*	SNPs and IN/DELs mostly in introns		

IN/DEL, insertion and deletion; SERT, serotonin transporter; SNP, single nucleotide polymorphism; VNTR, variable number of tandem repeat.

In addition to its role as a neuromodulator, there is increasing evidence that serotonin acts as a signaling molecule involving many aspects of neural development such as regulating cell proliferation, neuronal differentiation, neurite outgrowth, and synaptogenesis in mammals (Daubert and Condron, 2010; Wirth et al., 2017; Whitaker-Azmitia, 2020). In birds, serotonin levels in the brain and expression levels of serotonergic system-related genes during the development of serotonergic neurons have been clarified (Huang X. et al., 2019), and it has been shown that injecting serotonin externally to embryos affects neurodevelopment in chickens (Huang et al., 2021). As Huang et al. (2021) pointed out, in contrast to mammals that receive serotonin from the placenta during development, birds develop independently within eggs. Therefore, avian embryos may provide a unique model to investigate the role of serotonin as a morphogen in neurogenesis.

## 6. Conclusion and future perspectives

The anatomical organization of the avian central serotonergic system has been noted to be highly similar within vertebrates. In this review, we highlight that the molecular properties of the avian serotonergic system are also similar to those of the mammalian serotonergic system, supporting evolutionary conservation of the central serotonergic system in vertebrates. Going forward, it will be important to understand in detail the projection destinations of avian serotonergic neurons and to elucidate the functions involved at the neural circuitry level. Optogenetics, a revolutionary method for demonstrating the functions involved at the neural circuitry level, is becoming available in birds using viral vectors (Roberts et al., 2012; Rook et al., 2021). By combining such viral vectors with avian transgenic technology (Motono et al., 2010; Tsujino et al., 2019; Hagihara et al., 2020), it will be possible not only to comprehensively visualize targeted neural projection relationships, but also to elucidate the functions of avian neural circuits at the cellular level. Birds have emerged as unique model organisms for understanding the evolutionary continuity of the neural circuits responsible for cognition, behavior, and emotion. In addition to understanding the detailed correspondence between avian and mammalian brain structure, and between avian behavior assay systems and mammalian behavior, a neural circuitry level understanding of avian behavior regulated by the central serotonergic system could provide an opportunity for comparison with the neural circuits revealed in mammals. Such comparison of the neural circuits responsible for cognition, behavior, and emotion between birds and mammals will facilitate our understanding of the evolutionary continuity of the neural circuits.

## Author contributions

TF and SY designed the study and prepared the first draft, figures, and table. TF, NA, CM, KH, and SY wrote the paper. All authors have reviewed and accepted the final version of the manuscript.

## Funding

This work was supported by a Teikyo University Research Encouragement Grant (TF), Grants-in-Aid for Scientific Research from the Japan Society for the Promotion of Science (SY, 24590096, 15K07945, 18K06667; NA, 24790089, 20K06915; CM, 20K16472 and KH, 26440182, 17K07492, 20K06747), the Uehara Memorial Foundation (SY), the Sagawa Foundation for Promotion of Cancer Research (SY), and a Grant-in-Aid for Scientific Research on Innovative Areas “Memory dynamism” (26115522), “Adaptive circuit shift” (15H01449), and “Evolinguistics” (20H05012) from the Ministry of Education, Culture, Sports, Science, and Technology (KH), the Naito Foundation (KH), and the Japan Foundation for Applied Enzymology (KH).

## Conflict of interest

The authors declare that the research was conducted in the absence of any commercial or financial relationships that could be construed as a potential conflict of interest.

## Publisher’s note

All claims expressed in this article are solely those of the authors and do not necessarily represent those of their affiliated organizations, or those of the publisher, the editors and the reviewers. Any product that may be evaluated in this article, or claim that may be made by its manufacturer, is not guaranteed or endorsed by the publisher.
